# BADASS: BActeriocin-Diversity ASsessment Software

**DOI:** 10.1186/s12859-022-05106-x

**Published:** 2023-01-20

**Authors:** Sávio S. Costa, Gislenne da Silva Moia, Artur Silva, Rafael A. Baraúna, Adonney Allan de Oliveira Veras

**Affiliations:** 1 Parque de Ciência e Tecnologia Guamá, Laboratório de Engenharia Biológica, Belém, Pará Brazil; 2 Faculty of Computer Engineering, Federal University of Pará, Campus Tucuruí (CAMTUC-UFPA), Belém, Pará Brazil; 3 Faculty of Computing, Federal University of Pará, Campus Castanhal (FACOMP/CCAST), Belém, Pará Brazil

**Keywords:** Antimicrobial peptides, Bacteriocin, Metagenome mining, Software development

## Abstract

**Background:**

Bacteriocins are defined as thermolabile peptides produced by bacteria with biological activity against taxonomically related species. These antimicrobial peptides have a wide application including disease treatment, food conservation, and probiotics. However, even with a large industrial and biotechnological application potential, these peptides are still poorly studied and explored. BADASS is software with a user-friendly graphical interface applied to the search and analysis of bacteriocin diversity in whole-metagenome shotgun sequencing data.

**Results:**

The search for bacteriocin sequences is performed with tools such as BLAST or DIAMOND using the BAGEL4 database as a reference. The putative bacteriocin sequences identified are used to determine the abundance and richness of the three classes of bacteriocins. Abundance is calculated by comparing the reads identified as bacteriocins to the reads identified as 16S rRNA gene using SILVA database as a reference. BADASS has a complete pipeline that starts with the quality assessment of the raw data. At the end of the analysis, BADASS generates several plots of richness and abundance automatically as well as tabular files containing information about the main bacteriocins detected. The user is able to change the main parameters of the analysis in the graphical interface. To demonstrate how the software works, we used four datasets from WMS studies using default parameters. Lantibiotics were the most abundant bacteriocins in the four datasets. This class of bacteriocin is commonly produced by *Streptomyces* sp.

**Conclusions:**

With a user-friendly graphical interface and a complete pipeline, BADASS proved to be a powerful tool for prospecting bacteriocin sequences in Whole-Metagenome Shotgun Sequencing (WMS) data. This tool is publicly available at https://sourceforge.net/projects/badass/.

## Background

Characterization of bioactive molecules produced by free-living microorganisms has been very important in recent years because of their biotechnological applications. It is well known that the overwhelming majority of free-living microorganisms are not capable of being grown in laboratory conditions [1], which is a bottleneck to the identification and isolation of bioactive compounds. Thus, an alternative to search for new compounds is the well-established method of WMS, where the nucleic acids of the microbial community are extracted and sequenced directly from environmental samples [2]. Thus, genes involved in the synthesis of peptides or non-peptides bioactive compounds can be assessed. The main bottleneck lies in the development of user-friendly tools that allow the user to analyze a large amount of data in a simple and interactive way.

Several genes and molecules were prospected by WMS such as amylolytic or cellulolytic enzymes [3], antimicrobial compounds [4], antibiotic resistance genes (ARGs) [5–7] and bacteriocins [8]. Bacteriocins are small cationic thermostable bacterial peptides with a narrow spectrum of activity [9–11]. Unlike antibiotics, bacteriocins are produced by ribosomal activity and therefore are protease-sensitive. They are divided into three classes according to their synthesis mechanism.

Class I consists of peptides that after their translation, undergo structural changes. This class is also called lantibiotics. They have a molecular weight below 5 kDa and a size smaller than 28 amino acids [12, 13]. Class II is characterized by peptides that do not undergo post-translational modifications. They are larger than class I bacteriocins and have a molecular weight below 10 kDa [14]. Class III is composed of peptides with a molecular weight higher than 30 kDa. Bacteriocins of this class have a mechanism of action different from the other two classes, eliminating bacterial cells through cell wall hydrolysis [12, 15, 16].

A variety of software has been developed to search for ARGs or secondary metabolites such as nonribosomal peptide synthase (NRPS) or polyketide synthase (PKS) in WMS data [6, 17]. However, none of this software is focused on the prospecting of bacteriocins. Anti-SMASH [18] is an excellent tool to analyze genomes while RiPPER [19] works better for pan-genome data. BAGEL web tool (BActeriocin GEnome mining tooL) [17] is one of the first tools developed for the identification of peptides and bacteriocins in genome data. However, the tool has a maximum size for the input file, making difficult the analysis of WMS data.

In this article, we present BADASS software (BActeriocin-Diversity ASSessment Software), an automated pipeline with an intuitive graphical interface that allows users to analyze the diversity of bacteriocins using WMS raw data. Diversity measurement is based on the abundance and richness of the three bacteriocin classes currently described. The software is available at https://sourceforge.net/projects/badass/.

## Implementation

### Pipeline

**Fig. 1:**
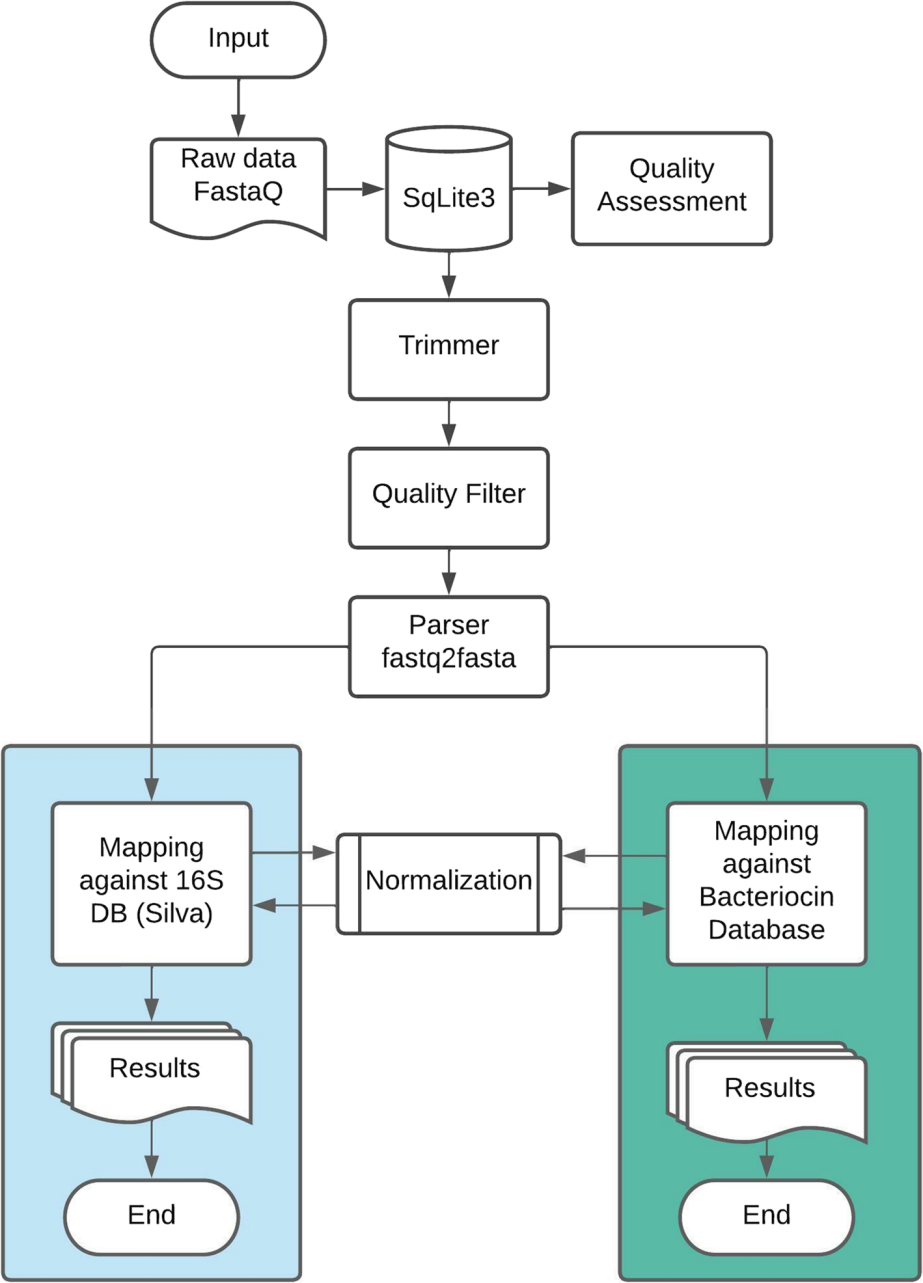
Diagram of the software pipeline

The pipeline of BADASS (Fig. 1) starts with the automatic loading of bacteriocin sequences to the database. It is worth noting that this process needs to be executed only on the first use of the software. The input file consists of a WMS sequencing sample in FASTQ format. After saving the project the following steps are performed.

- *Quality assessment* The user can choose to evaluate the raw data with a boxplot chart that correlates the Phred score of a base (y axis) with base position (x axis). This is an optional step that helps users to decide about the quality filter values that will be used in the next step. A boxplot with the result of the FastQC analysis is produced at this stage and displayed to the user.
- *Trimming and quality filter* Raw data is trimmed to remove bases at the end of the reads with a Phred score below the cut-off value provided by the user. Subsequently, sequences are filtered according to parameters such as alignment score and e-value. The Fastx Toolkit software is used in this step.
- *Parser fastq2fasta* The trimmed and filtered file is converted into FASTA format.
- *Mapping against the bacteriocin database* In order to identify bacteriocins, we adapted a search method used in several works [20, 21] which consists of: Firstly, a database is built using non-redundant BAGEL4 sequences [22]. Subsequently, the BLAST+ tool [22, 23] is used to compare the translated reads against the BAGEL4 database with blastx. The best hit for each read identified as bacteriocin is retrieved. The user can define an e-value cut-off for the homology analysis.
- *Mapping against the 16S rRNA SILVA database* The same file of the previous step is used to align the reads against the SILVA database using DIAMOND [24]. Two files in.csv format are generated. The first contains the list of subject nucleotide sequences with their respective identity values. The other file contains the best hit for each query sequence based on an identity cut-off value provided by the user. Cut-off values are adjustable in the graphical interface of BADASS.

The number of reads identified as bacteriocins and 16S rRNA are used to calculate the richness and abundance of bacteriocins in the WMS dataset as mentioned later.

### Programming language and database

BADASS was developed in JAVA (https://www.oracle.com) and used the Maven tool (https://maven.apache.org/) to build and manage the project. Maven was used due to the automated management and generation of the JAR package containing the software dependencies. Swing library was used to produce the graphical interface. The database management system used to control the steps and manage the project was SQLite v.3 (https://www.sqlite.org/).

### Data source and software validation

The software validation was performed using four whole-metagenome shotgun sequencing datasets. Samples were obtained in the Tucuruí Hydroelectric Power Plant water reservoir submitted in EBI database under the accession numbers ERS1560860, ERS1560861, and ERS1562591 [8] and a sample obtained from Unai's Hot Spring from the ENA (European Nucleotide Archive) database with the accession number PRJEB8864. The following parameters were used: Quality threshold: 20, minimum length: 100, minimum quality score to keep: 16, minimum percent: 80%, e-value: 10, threads: 6, identity: 50.

### Quality assessment and BLAST

BADASS uses the statistical platform R v.4 (https://www.r-project.org) for quality assessment of the raw data, through the FastQC package (https://github.com/kassambara/fastqcr). Reads are trimmed and quality filtered using the FASTX-Toolkit (http://hannonlab.cshl.edu/fastx_toolkit/). The user is able to adjust the parameters of quality filtering in the graphical interface of BADASS. BLAST v.2.0.9-3 and Diamond v.2.0.7.145 software are used to map the reads against the BAGEL4 [22, 25] and SILVA [26] databases, respectively. BAGEL4 was updated and now has about 500 Ribosomally synthesized and post-translationally modified peptides (RiPPs) (class 1), 230 non-modified bacteriocins (class 2), and 90 bacteriocins with more than 30 kDa (class 3). RippMIner [27] and the data repository MIBiG [28] were used to build the BAGEL4 database.

### Abundance analysis

Diversity of bacteriocins was analyzed in terms of abundance and richness. In order to calculate the abundance of bacteriocins we adapted the formula proposed formula in studies involving the search of abundance of resistance genes in WMS data. [20]. Where: (1) *n* is the amount of bacteriocins that were found in sample; (2).*N*_*bacteriocin sequences*_ is the number of reads that mapped to a specific bacteriocin; (3) *T*_*read*_ is the average size of reads; (4) *T*_*bacteriocin*_ is the average size of the bacteriocin; (5) *N*_*16S rRNA sequences*_ is the number of reads that mapped to 16S rRNA sequences; and (6) *T*_*16S rRNA sequence*_ is the average size of the 16S rRNA sequence.

$$Abundance = \mathop \sum \limits_{1}^{n} \frac{{N_{bacteriocinsequences} \times \frac{{T_{read} }}{{T_{bacteriocin} }}}}{{N_{16SrRNAsequences} \times \frac{{T_{read} }}{{T_{16SrRNA} }}}}.$$

### Workstation

Analyses were performed in a Desktop equipment Intel® Core™ i7-10510U CPU @ 1.80 GHz with 8 processing cores, 16 GB of RAM memory, and tests were run on Ubuntu 21.10, 64-bit, Windows 11 and macOS Ventura 13.0 operating systems.

## Results and discussion

BADASS was developed using the BLAST and DIAMOND alignment tools to identify bacteriocin and 16S rRNA sequences in WMS raw data. The choice of tool can be defined in the BADASS GUI. Other studies have used similar methodologies for prospecting relevant genes such as ARGs [7, 20, 29, 30]. For example, ARGs-OAP is an online pipeline for antibiotic resistance genes detection in metagenomic data through similarity sequence analysis [5]. In addition to the homology search, BADASS calculates the abundance of each bacteriocin class by taking into account the size of the genes and the size of the reads produced by the sequencing library [22]. The number of reads identified as bacteriocins are compared to the number of reads identified as the gene of the 16S rRNA, which is present in a few copies per cell. This approach makes the size of the reads as well as the size of the genes not interfere with the analysis of abundance. Thus, this pipeline is a powerful tool for rapid and comprehensive evaluation of bacteriocin diversity using WMS raw data.

The main results of BADASS are the description of richness and the values of the abundance of bacteriocins using WMS raw data as input file in a simple and intuitive way. The software provides a set of adjustable parameters in the graphical interface. Users can also choose to process the samples using default parameters. In more detail, the results obtained by the software include: (1) quality assessment box plots of the raw data directly in the graphical interface or even in the results folder; (2) spreadsheets in.xlsx or.csv format containing information about the identified bacteriocins (richness) including their frequency (ratio between the number of reads identified as bacteriocin and the total number of reads in a sample) and abundance (calculated using the formula previously mentioned), organized by class; (3) bar plots of abundance based on the.csv files; (4) a.csv file containing the list of 16S rRNA sequences identified in the dataset including the value of percentage identity; (5) Trimmed.fastq and QV.fastq files containing the trimmed reads and the reads filtered by minimum size, respectively.

It is also possible to detect bacteriocin in genome sequences using other software. Table 1 presents several computational tools and databases developed to help in the identification of these antimicrobial peptides. The main features of each software or database are compared in the table. It is worth noting that BADASS is the only who has a graphical interface, supports WMS data, and performs diversity analysis (Table 1). BAGEL4 stands out for having one of the most complete databases containing a large number of annotated and experimentally verified bacteriocin sequences. In addition, the database is divided into three classes according to the genetic information and mechanism of action. Because of these features, BAGEL4 was used as a reference bacteriocin database in BADASS. BACTIBASE [30] is a database containing detailed information about the physicochemical properties of bacteriocins. This information allows a fast and accurate prediction of the structure–function relationship and possible target organisms of the antimicrobial peptides. Other relevant software includes BOA (Bacteriocin Operon Associator) [31] which uses Hidden Markov Models to predict bacteriocin clusters, Neubi [32] which identifies bacteriocins using a word embedding approach, and Anti-SMASH (Antibiotics and Secondary Metabolite Analysis Shell) which was launched in 2011 and is used not only for bacteriocin prediction but for a number of other secondary metabolites [18, 33].

**Table 1:** Comparison of the main features of software used for bacteriocin gene mining

Software	Bacteriocin database integrated	Bacteriocin detection	Open-source and stand-alone available	Friendly graphical interface	Detection of other biosynthetic classes	Search in metagenome data	Search standardization through data normalization	Search flexibility
BADASS	**+**	**+**	**+**	**+**	**+**	**+**	**+**	**+**
BOA	**+**	**+**	**+**	**−**	**−**	**−**	**−**	**+**
BACTIBASE	**+**	**−**	**−**	**−**	**+**	**−**	**−**	**−**
BAGEL	**+**	**+**	**+**	**−**	**+**	**−**	**−**	**−**
antiSMASH	**+**	**+**	**+**	**−**	**+**	**−**	**−**	**−**
Neubi	**+**	**+**	**+**	**−**	**−**	**−**	**−**	**+**

A plus sign indicates that the feature is available

The identification of bacteriocins, however, is still quite challenging due to the limited number of known and experimentally analyzed sequences. Choosing the most appropriate and up-to-date tool is essential for the search and identification of bacteriocin genes. BADASS is a user-friendly software, with a robust pipeline that starts with the quality assessment of the raw data and ends with the analysis of the richness and abundance of bacteriocins.

A pilot analysis was performed using four datasets with default parameters. The results of the dataset ERR1816708 are presented in Table 2. First column of the table shows the BAGEL4 accession number and name of the bacteriocins. Other columns correspond to frequency, abundance and class, respectively. Thus, users are able to identify the diversity of bacteriocins in the dataset. Additionally, complementary analyses such as taxonomic affiliation of the microbial community are important to determine the ecological context of the described bacteriocins [8].

**Table 2:** CSV file generated by the BADASS presenting the names and other information of the detected bacteriocins

Bacteriocin name	Frequency	Abundance	Class
SED43766.1 hypothetical protein SAMN05428943_3617 [Streptomyces sp. 2314.4]	3543	0032527	class1
CAX48972.1 labyrinthopeptin A1/A3 prepropeptide [*Actinomadura namibiensis*]	2143	0021642	class1
tr|A0A0Y0LSJ1|A0A0Y0LSJ1_STREE PldA1 OS = *Streptococcus pneumoniae* OX = 1313 PE = 4 SV = 1	4565	0030734	class1
WP_043998581.1 microcyclamide/patellamide family RiPP [*Microcystis aeruginosa*]	5007	002593	class1
AAL73241.1 LanA [*Streptococcus mutans*]	4195	0026898	class1
AAL15567.1 lantibiotic ericin Sa [*Bacillus subtilis*]	3397	0024504	class1
tr|I6XG59|I6XG59_STAAU Lantibiotic OS = *Staphylococcus aureus* OX = 1280 PE = 3 SV = 1	3584	0030803	class1
tr|D2K7B5|D2K7B5_9NOST Anacyclamide OS = Anabaena sp. SYKE 763A OX = 701,084 GN = acyE PE = 4 SV = 1	2438	0020099	class1
sp|E9K9Z1|GCCF_LACPN Bacteriocin glycocin F OS = *Lactobacillus plantarum* OX = 1590 GN = gccF PE = 1 SV = 1	3193	0020153	class1
sp|P83375|BSP43_SERPL Bacteriocin serracin-P 43 kDa subunit (Fragment) OS = *Serratia plymuthica* OX = 82,996 PE = 1 SV = 2	189	0008482929	class2
NP_268769.1 conserved hypothetical protein—bacteriocin like peptide associated [*Streptococcus pyogenes* M1 GAS]	2049	0010218414	class2
AAC95138.1 bacteriocin [*Brochothrix campestris*]	1976	0010366276	class2
AAO18426.1 plantaricin NC8 beta peptide precursor [*Lactobacillus plantarum* subsp. plantarum NC8]	3225	0023686101	class2
sp|P86183|ETCHF_ENTFC Enterocin-HF OS = *Enterococcus faecium* OX = 1352 GN = entHF PE = 1 SV = 2	2332	0016241536	class2
tr|O54454|O54454_STRTR Amphipathic pore-forming peptide OS = *Streptococcus thermophilus* OX = 1308 GN = thmA PE = 4 SV = 1	3208	0015245511	class2
ACR43770.1 lactococcin G beta peptide [*Lactococcus lactis*]	2727	0018359483	class2
AAL77872.1 Leucocin K [*Lactobacillus paraplantarum*]	3096	0024050503	class2
ZP_01821307.1 bacteriocin BlpM [*Streptococcus pneumoniae* SP6-BS73]	2962	0014244012	class2
CAA61099.1 colicin K [*Escherichia coli*]	15481	001141156	class3
sp|P09883.4|CEA9_ECOLX RecName: Full = Colicin-E9	23591	0016373817	class3
CAA44310.1 bacteriocin 28b [*Serratia marcescens*]	24094	0021676498	class3
prf||1003181A colicin A 20kd fragment	6302	0012298003	class3
pdb|1CII| Colicin Ia	16010	0010742897	class3
YP_366690.1 Linocin_M18 bacteriocin protein [*Burkholderia lata*]	11076	0016509739	class3
AAT85003.1 klebicin C phage associated protein [*Klebsiella pneumoniae*]	21117	0022447872	class3
sp|P02978|CEA1_ECOLX Colicin-E1 OS = *Escherichia coli* OX = 562 GN = cea PE = 1 SV = 1	2042	0001580199	class3
AAA59418.1 colicin protein (plasmid) [*Escherichia coli*]	9672	0007484665	class3

**Fig. 2:**
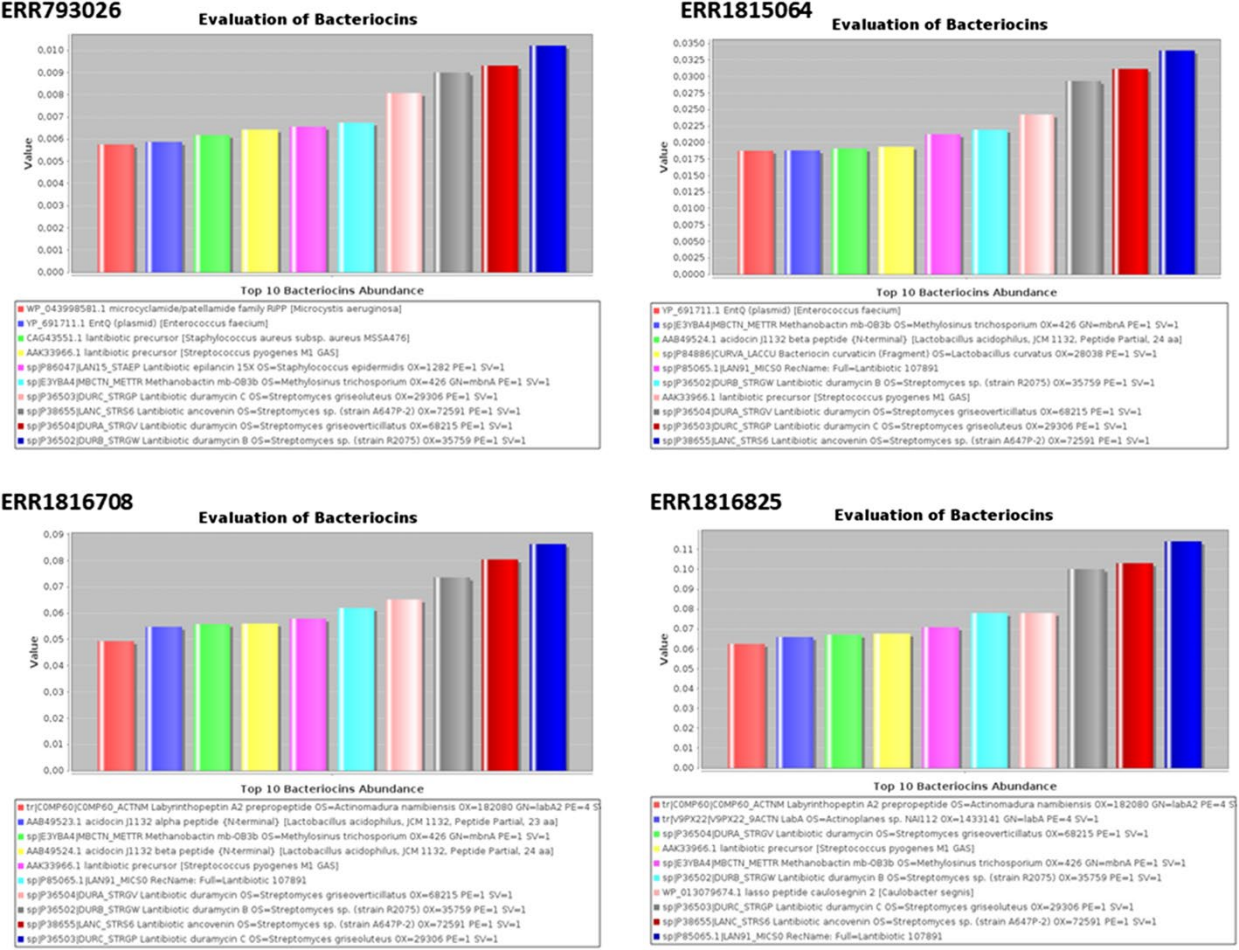
Output file of BADASS. Bar plots for the four datasets analyzed presenting the top ten most abundant bacteriocins

**Fig. 3:**
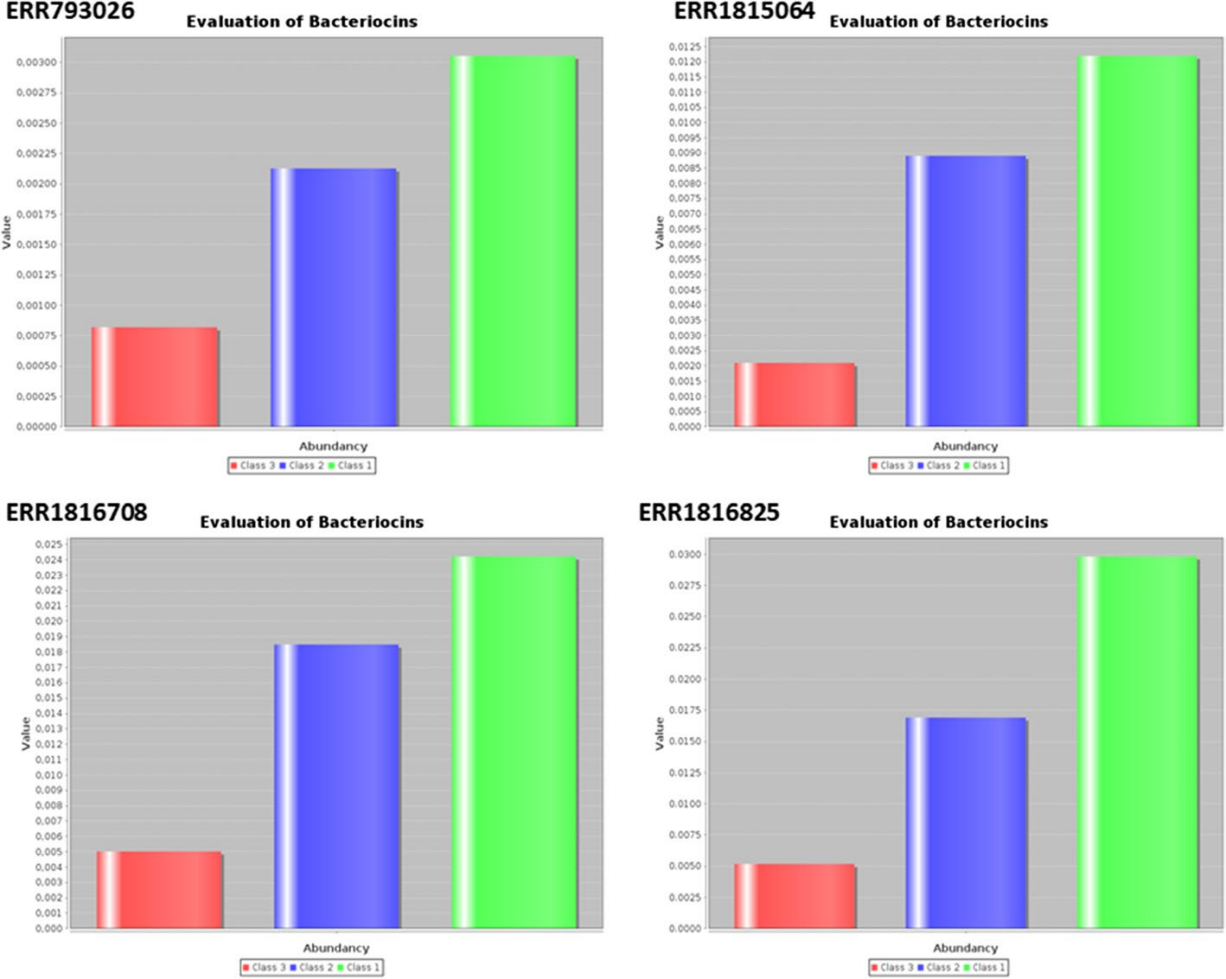
Output file of BADASS. Bar plots presenting the abundance of each class of bacteriocin in the four datasets analyzed

Two bar plots were generated by the software containing an overview of the bacteriocin diversity. The first plot (Fig. 2) is designed based on a.csv file similar to Table 2. The plot presents the top ten most abundant bacteriocins in the dataset. Information about the bacterial species that commonly produce the peptides are presented in the legend. The second plot (Fig. 3) presents the abundance of bacteriocins by class. The best parameters for each study should be carefully chosen by the user according to it dataset characteristics. A variation in the results is expected since the parameters adjust the analysis performed by the software. The choice of parameters by the user will result in changes in the result, being able to restrict to stricter or looser parameters [34].

The time required for similarity search and post-alignment analysis has become a bottleneck as sequence costs decrease and the size of the datasets increases [35]. We also highlight that all the analysis, starting from the filtering of the raw data can be done in the BADASS pipeline. The software allows users to modify most of the parameters such as e-value, identity cut-off, and others.

## Conclusions

In the environment, a countless number of microbial species coexist and, in order to succeed in colonize their ecological niches, many have developed mechanisms to eliminate other species through the production of antimicrobial molecules. In this chemical warfare, bacteriocins are narrow-spectrum antimicrobial peptides synthesized by ribosomal activity that are widely distributed in bacterial species. Thus, the development of computational tools to identify, classify and quantify bacteriocins in WMS datasets is of great importance for microbial ecology and biotechnology.

BADASS provides the user with a robust and automated computational tool with a simple and intuitive graphical interface, where the parameters can be adjusted by the user, allowing greater independence in the analysis of different samples. The integration of the software with the R statistical platform allows the generation of plots that helps in data interpretation. For those looking to prospect antimicrobial peptides in WMS raw data, BADASS is a powerful solution.

## Availability and requirements

Project name: BADASS

Project home page: https://sourceforge.net/projects/badass/

Operating system(s): platform independent

Programming language: Java

Other requirements: e.g. Java 19.0.1 or higher

License: GNU GPL

Any restrictions to use by non-academics: license needed.

## Data Availability

The software is available at https://sourceforge.net/projects/badass/ and samples were obtained in the Tucuruí Hydroelectric Power Plant water reservoir submitted in EBI database under the accession numbers ERS1560860, ERS1560861, and ERS1562591 and a sample obtained from Unai's Hot Spring from the ENA (European Nucleotide Archive) database with the accession number PRJEB8864.

## Abbreviations

BADASS: BActeriocin-Diversity ASsessment Software
WMS: Whole-Metagenome Shotgun Sequencing
ARGs: Antibiotic resistance genes
NRPS: Nonribosomal peptide synthase
PKS: Polyketide synthase
BAGEL: BActeriocin GEnome mining tool
ENA: European Nucleotide Archive
RiPPs: Ribosomally synthesized and post-translationally modified peptides
